# 887. Associated factors with Monkeypox in patients with suspected infection in a university hospital in Colombia.

**DOI:** 10.1093/ofid/ofad500.932

**Published:** 2023-11-27

**Authors:** Santiago Arboleda, Fabian Mantilla, Elizabeth Reyes, Sandra Valderrama, Carlos Alvarez

**Affiliations:** Hospital universitario San Ignacio, Bogotá, Distrito Capital de Bogota, Colombia; Pontificia Universidad Javeriana, Bogotá, Distrito Capital de Bogota, Colombia; Pontificia Universidad Javeriana, Bogotá, Distrito Capital de Bogota, Colombia; Pontificia Universidad Javeriana, Bogotá, Distrito Capital de Bogota, Colombia; Universidad Nacional de Colombia, Bogotá, Distrito Capital de Bogota, Colombia

## Abstract

**Background:**

Monkeypox was an infection limited to the African continent, the study of which regains relevance after the breaking of its geographical barrier in May 2022. In Colombia, in agreement to the Colombian National Institute of Health 4090 cases have been confirmed and 97% have occurred in men. We evaluate epidemiological and clinical characteristics of people suspected of being diagnosed with monkeypox and we compare the differences between patients who underwent positive RT-PCR tests with those who had negative results in order to determine clinical variables that increase the possibility of having a positive result.

**Methods:**

This is a cross-sectional case-control study between August and December 2022 in adult patients who consulted due to suspected Monkeypox infection in a university hospital in Bogota-Colombia. We characterized the different epidemiological and clinical characteristics of the disease. We performed an exploratory analysis of the variables related to a positive RT-PCR for Monkeypox. The cases were patients with positive RT-PCR and the controls were patients with a negative result. A multivariate analysis was carried out by unconditional logistic regression and the variables with a p< 0.2 and that did not present collinearity were included in the model. All analyses were conducted in R software version 4.1

**Results:**

A total of 162 medical records of patients with suspected diagnosis of smallpox were analyzed. 102 samples were RT-PCR positive for monkeypox and 60 were negative. Table 1 describes the bivariate analysis. All patients who had positive RT-PCR were MSM. In the multivariate model the associated factors with having a positive RT-PCR test for Monkeypox were: age (OR:0.01, CI95%:0.01-0.17), contact with a suspected or confirmed case (OR:6.08, CI95%:1.96-21.65), fatigue (OR:3.91, CI95%:1.47-11.43), arthralgia (OR:7.45, CI95%:1.48-58.41), lymphadenopathy (OR:10.46, CI95%:3.50-36.44) and perianal lesions (OR:18.64, CI95%:4.64-104.53).

**Figure:**
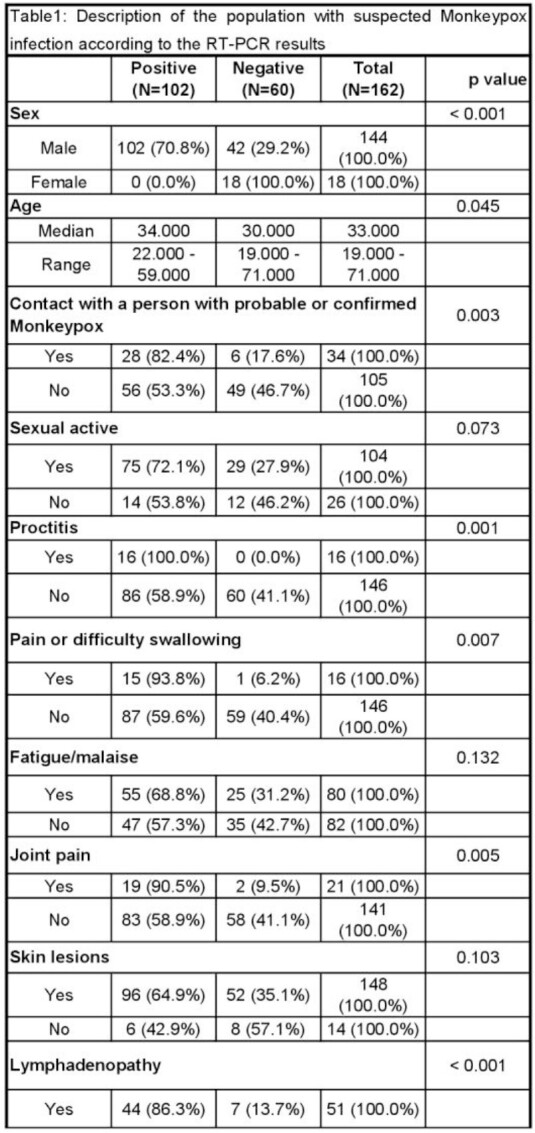

Part1

**Figure:**
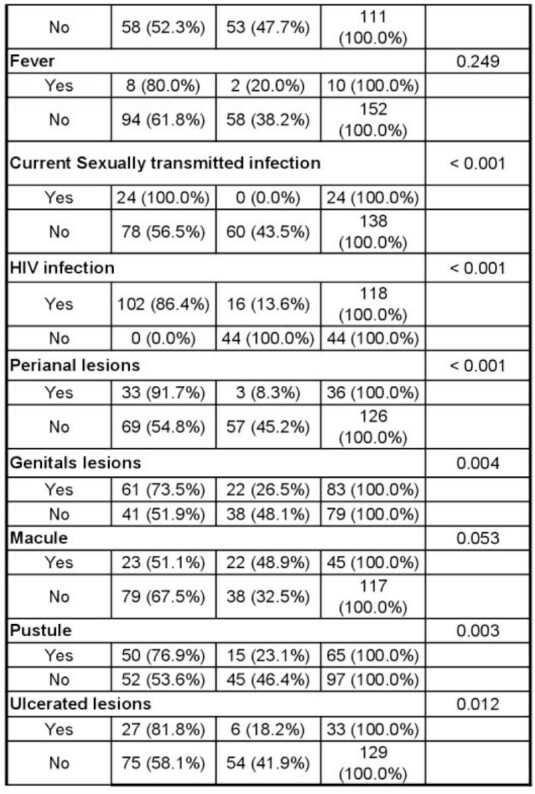

Part 2

**Conclusion:**

This study reaffirms the increased frequency of monkeypox infection in MSM and HIV-positive patients. In patients who consult for suspected monkeypox, there is an increased odds of infection in patients with contact with Monkeypox, fatigue, arthralgia, lymphadenopathy or perianal lesions.

**Disclosures:**

**All Authors**: No reported disclosures

